# A High Force of *Plasmodium vivax* Blood-Stage Infection Drives the Rapid Acquisition of Immunity in Papua New Guinean Children

**DOI:** 10.1371/journal.pntd.0002403

**Published:** 2013-09-05

**Authors:** Cristian Koepfli, Kathryn L. Colborn, Benson Kiniboro, Enmoore Lin, Terence P. Speed, Peter M. Siba, Ingrid Felger, Ivo Mueller

**Affiliations:** 1 Swiss Tropical and Public Health Institute, Basel, Switzerland; 2 University of Basel, Basel, Switzerland; 3 Walter and Eliza Hall Institute, Parkville, Victoria, Australia; 4 University of California, Berkeley, Department of Biostatistics, Berkeley, California, United States of America; 5 Papua New Guinea Institute of Medical Research, Goroka, Eastern Highland Province, Papua New Guinea; 6 Barcelona Centre for International Health Research, Barcelona, Spain; Queensland Institute for Medical Research, Australia

## Abstract

**Background:**

When both parasite species are co-endemic, *Plasmodium vivax* incidence peaks in younger children compared to *P. falciparum*. To identify differences in the number of blood stage infections of these species and its potential link to acquisition of immunity, we have estimated the molecular force of blood-stage infection of *P. vivax* (_mol_FOB, i.e. the number of genetically distinct blood-stage infections over time), and compared it to previously reported values for *P. falciparum*.

**Methods:**

*P. vivax*_mol_FOB was estimated by high resolution genotyping parasites in samples collected over 16 months in a cohort of 264 Papua New Guinean children living in an area highly endemic for *P. falciparum* and *P. vivax*. In this cohort, *P. vivax* episodes decreased three-fold over the age range of 1–4.5 years.

**Results:**

On average, children acquired 14.0 new *P. vivax* blood-stage clones/child/year-at-risk. While the incidence of clinical *P. vivax* illness was strongly associated with *_mol_*FOB (incidence rate ratio (IRR) = 1.99, 95% confidence interval (CI95) [1.80, 2.19]), _mol_FOB did not change with age. The incidence of *P. vivax* showed a faster decrease with age in children with high (IRR = 0.49, CI95 [0.38, 0.64] p<0.001) compared to those with low exposure (IRR = 0.63, CI95[0.43, 0.93] p = 0.02).

**Conclusion:**

*P. vivax*_mol_FOB is considerably higher than *P. falciparum*_mol_FOB (5.5 clones/child/year-at-risk). The high number of *P. vivax* clones that infect children in early childhood contribute to the rapid acquisition of immunity against clinical *P. vivax* malaria.

## Author Summary

In areas where *P. vivax* and *P. falciparum* parasite species co-occur, immunity to *P. vivax* seems to be acquired more rapidly. This difference could be caused either by generic differences in the way immunity is acquired or by a relatively higher exposure to *P. vivax* blood-stage infections in early life. We found that children experienced an average of 14 new *P. vivax* blood-stage infections per year, and that the number of new infections acquired predicted how often children fell ill with vivax malaria by genotyping all *P. vivax* infections that occurred in a group of 264 children 1–4 years of age followed for 16 months. The burden of blood-stage infections caused by *P. vivax* was therefore at least twice as high as that caused by *P. falciparum*. This higher force-of-blood-stage infection (_mol_FOB) caused by *P. vivax* is at least partially due to the ability of *P. vivax* hypnozoites to relapse from long-lasting liver stages. A high exposure to *P. vivax* blood-stage infection resulted in more rapid decrease in the incidence of *P. vivax* malaria. The high number of *P. vivax* clones that infect children in early childhood is thus likely to contribute substantially to the rapid acquisition of immunity against clinical *P. vivax* malaria.

## Introduction

Exposure to malaria – i.e. the number of blood-stage infections acquired over time – determines to a large extent the frequency of disease and can also explain seasonal patterns and age trends. The number of infections acquired over time depends on transmission intensity, with frequent bites by infected mosquitoes resulting in high numbers of infections in a short period of time. In *P. vivax*, additional blood-stage infections are caused by relapses from dormant hypnozoites.

As people who live in malaria endemic areas achieve immunity to disease after several years of exposure, newly acquired infections do not always result in clinical episodes. The speed of acquisition of immunity depends on transmission intensity [1]–[3] and also differs between parasite species, with immunity to *P. vivax* appearing to be acquired faster than immunity to *P. falciparum* [4], [5]. In numerous field studies conducted in areas co-endemic for both species, the burden of *P. vivax* infections and disease was found to peak at a younger age than that of *P. falciparum* [6]–[10].

The mechanisms underlying the immunity to malaria are not entirely understood [2], [11]. Whereas semi-immune people still develop blood stage parasitemia, parasite densities are considerably lower and rarely cause fever. Differences in the rate of natural acquisition of immunity to *P. falciparum* and *P. vivax* may be the result of differences in the immune responses induced by either species, and/or a consequence of different numbers of infections acquired over time.

Malaria infections consist of different parasite clones that can infect individuals successively or simultaneously as multiple clone infections. Clones differ in their polymorphic surface antigens as well as in neutral genetic markers. Genotyping of such markers allows discrimination of individual parasite clones during multiple clone infections. The diversity of antigens is well documented in *P. falciparum* [12]–[14] and *P. vivax* [15], [16]. The large number of antigen variants is thought to allow parasites to escape the immune system, as new infections likely express alleles different from previous clones, and thus are not recognized by the present humoral response [17], [18]. Immunity is thus assumed to be largely clone-specific, providing little cross-protection against heterologous clones [4], [19]–[22].

*Plasmodium falciparum* clones transmitted by a mosquito bite appear in the blood stream within 7–10 days. Albeit some of the transmitted sporozoites might not establish blood-stage infections, the number of genetically distinct clones detected in the blood-stream over time (_mol_FOB, as determined by genotyping) is a direct measure of the molecular force of infection (_mol_FOI, defined as number of distinct clones entering the body over time). It is thus closely linked to intensity of transmission [23].

In tropical and sub-tropical areas, *P. vivax* infectious mosquito bites also lead to primary blood stage infections within 10–14 days. In addition, a proportion of parasites remain dormant in the liver. Relapses from such hypnozoites can then occur months later and result in delayed blood stage infections. In high transmission regions, such relapsing parasites are mostly genetically different from parasites detected during the last acute blood stage infection [24], [25]. The relapsing clones might have been present earlier as blood stage infection [26], or the primary and relapsing infections are genetically different clones that were jointly transmitted in a single mosquito bite with only one of these clones having emerged from the liver. Thus, relapses may not only boost existing immune responses by repeated exposure to the same parasite clone, but also lead to a broader immune repertoire. No genotyping approach allows differentiating a relapse from a primary blood stage infection. The number of *P. vivax* parasites detected in the peripheral blood in a given interval (_mol_FOB) is therefore a combination of primary blood-stage infections and relapses from the same and/or earlier mosquito bites.

In order to assess the association between (individual) exposure and risk of malaria, we followed 264 children aged 1 to 3 years at enrolment in an area of high endemicity of *P. falciparum* and *P. vivax* in Papua New Guinea (PNG) over 16 months [23], [27], [28]. As in earlier studies in PNG [6], [10], [29], *P. vivax* incidence in this cohort peaked in younger children compared to *P. falciparum* incidence [27]. While *P. vivax* incidence decreased throughout the age group, *P. falciparum* incidence increased between the ages of 1 to 3.5 years with little change thereafter [27]. Previously we had estimated the *P. falciparum*_mol_FOI by *msp2* genotyping, and shown that children acquired 5.9 new *P. falciparum* infections per year-at-risk [23]. *P. falciparum*_mol_FOI increased significantly with age and was highly predictive of incidence patterns [23].

The diversity and multiplicity of *P. vivax* in this cohort was published previously [28]. In this paper, we present estimates of the *P. vivax* molecular force of blood-stage infection (_mol_FOB).

## Methods

### Ethics statement

Informed written consent was obtained from all parents or guardians prior to recruitment of each child. Scientific approval and ethical clearance for the study was obtained from the Medical Research and Advisory Committee (MRAC 05.19 and 09.24) of the Ministry of Health in PNG and from the Ethikkommission beider Basel in Switzerland (no 03/06).

### Field survey and patients

This study was conducted in Ilaita, a rural area near Maprik, East Sepik Province, Papua New Guinea. A detailed description of the study was given elsewhere [27]. Briefly, 264 study participants were enrolled at an age of 10 to 38 months between March and September 2006, and followed actively every 2 weeks to determine malaria morbidity for a period of up to 16 months (until July 2007). In addition, children were actively checked every 8 to 9 weeks for the presence of malarial infections. Except for the first and last round of active case detection, two consecutive blood samples were collected by finger prick 24 hours apart from each study participant at each follow-up visit. An individual thus contributed up to 16 samples, 14 of which were paired samples collected 24 hours apart. A passive case detection system was maintained at the local health center and aid post throughout the entire study period. At each episode of febrile illness, a blood sample was collected from the participant and a rapid diagnostic test (RDT) was performed and haemoglobin measured. Antimalarial treatment with arthemeter-lumefantrine (AL) or in a few cases with amodiaquine plus sulphadoxine-pyrimethamine was administered upon a positive RDT or if haemoglobin levels were less than 7.5 g/dl. In children with negative RDT, blood slides were read within 24 hours, and microscopy positive children were treated with AL.

### Laboratory procedures

For genotyping individual *P. vivax* clones, the molecular markers *msp1*F3 and MS16 were typed using capillary electrophoresis for highly precise fragment sizing, which is a precondition for longitudinal follow up of individual parasite clones. Both markers proved to be highly polymorphic in the cohort with an expected heterozygosity (*He*) of 97.8% for MS16 and 88.1% for *msp1*F3. Details of the genotyping have been described previously [28].

### Data analysis

In a previous analysis of the samples collected 24 hours apart, we found that not all clones present in a host were detected within a single sample. Twenty-one percent of all *msp1*F3 alleles and 28% of all MS16 clones were missed on a single day [30]. Thus, we used the combined genotyping data from both day 1 and day 2, except for samples from enrolment and final visits, where only 1 venous blood sample was taken.

The force of new *P. vivax* blood-stage infections (_mol_FOB) was generated by counting the number of genotypes in each interval that had not been present in the preceding interval. An 8 to 9 weeks interval started on the first day after a regular cross-sectional visit and included all samples collected during passive case detection over two months plus the samples collected at the end of the interval. _mol_FOB was also determined for both markers combined, *msp1*F3 and MS16. In case of discrepancy between the markers for an 8-weeks interval the higher estimate from either marker was used. This approach corrected for imperfect resolution and detectability of a single marker.

Genotyping cannot directly identify relapses; _mol_FOB measures the combination of primary blood-stage infections and those caused by relapsing hypnozoites. Thus, homologous relapses occurring in two subsequent 2-month intervals would be misclassified as persisting clones. New Guinean *P. vivax* strains are known to relapse rapidly [31], however in regions of high transmission, relapsing clones are usually of a different genotype than the initial blood stage infection [24]. As a consequence the number of homologous relapses that were not detected is expected to be relatively small.

In line with the pharmacokinetic properties of the drugs [32]–[34], children were not considered at risk for two weeks after treatment with artemether-lumefantrine and four weeks after treatment with amodiaquine (AQ) plus sulphadoxine-pyramethamine (SP). The force of blood-stage infection for each child and interval was subsequently converted into the number of new clones acquired per year-at-risk.

Similar to previous analyses of *P. falciparum*_mol_FOI [23], generalized linear mixed models (GLMMs) were used for analyses of force of blood-stage infection as well as for incidence of *P. vivax* episodes. These models were chosen because they allowed the fixed effects to be specified separately from the random effects (i.e. repeated measurements from the same child over time and unmeasured village factors). Furthermore, the random-effects model allowed for decomposition of the error into between-village and within-village variation.

**Figure 1:**
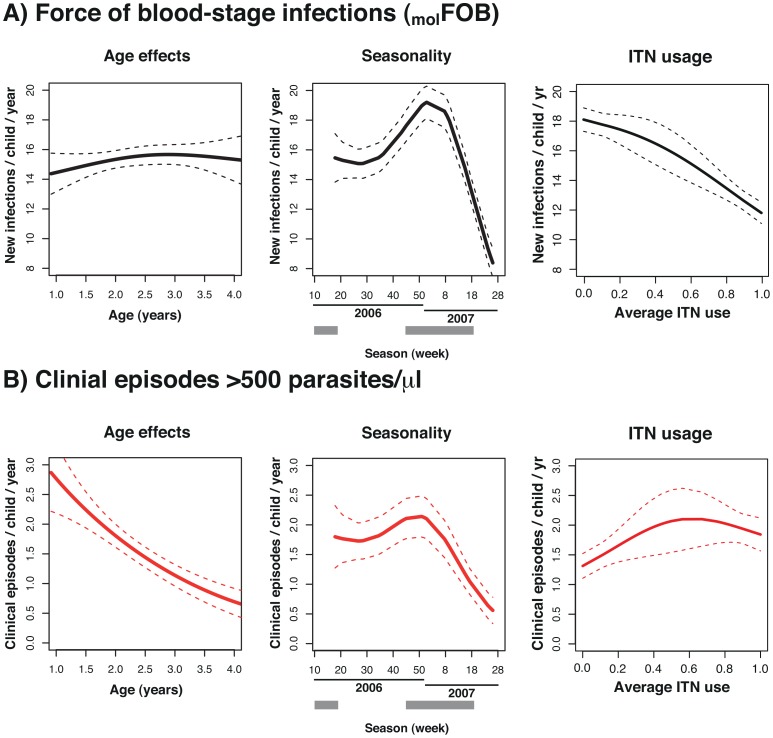
Age and seasonal patterns of molecular force of blood-stage infection (_mol_FOB, Panel A) and incidence of clinical *P.* *vivax* malaria (Panel B), and effects of ITN use on these parameters. Smooth splines from generalized additive models with single predictors and 95% confidence intervals.

We fit a Poisson GLMM model with a log link to relate the fixed and (Gaussian) random effects to the number of clinical episodes experienced during a two month interval (defined as febrile illness plus *P. vivax* >500 parasites/µl). Covariates were selected based on earlier analyses of the same data [27]. Seasonality was characterized by two readily interpretable parameters: the amplitude, which was half the range between the peak and trough, and the phase, which was the location of the first zero crossing in a cycle relative to the origin in time (in this case, the first week of the year). For computational convenience, they were replaced by sine and cosine terms with fixed phases. For all outcomes except prevalence, an offset was fit to adjust for years at risk. Estimation of these models was done using the LME4 package in R version 2.12 [35]. All point estimates provided throughout the text (except those for seasonal effects) were obtained from cubic splines fit using generalized additive models (Figure 1) using the MGCV package in R version 2.12 [36]. For a more detailed description of the statistical approaches see [23]. Point estimates for seasonal peaks and troughs were obtained from the GLMMs by setting all other values of the covariates at their means. For the analyses of the effect of exposure on the relationship between age and incidence of *P. vivax* malaria, children were stratified into terciles according to the average _mol_FOB during the entire follow-up.

## Results

A total of 264 children aged 0.9 to 3.2 years at baseline were enrolled and followed up for 69 weeks. Out of 264 children, 248 (93.9%) were retained until the end of the study with 96.0%–100.0% of children seen at each scheduled two-monthly survey. Over the entire follow-up period, the age ranged from 0.9 to 4.5 years. A detailed description of this cohort was published previously [27]. All but five children had at least one *P. vivax msp1*F3 or MS16 PCR positive sample during the 16 months of follow-up. Of all samples collected, 51.6% and 52.7% were positive for *msp1*F3 and MS16, respectively, and 54.8% were positive for either marker. In a total of 1448 *P. vivax* positive samples, 2,305 and 3,372 distinct clones were detected by *msp1*F3 (65 different alleles) and MS16 (113 alleles) genotyping, respectively.

### Force of blood-stage infection

Excluding any period with residual drug levels from the time at risk, each child was at risk of acquiring new infections for an average of 0.93 years during the cohort (95% confidence interval (CI_95_) [0.91, 0.96] range: 0.11–1.32). On average, 8.7 *P. vivax msp1*F3 (CI_95_ [8.1, 9.4], range: 0–30) and 12.8 MS16 clones (CI_95_ [11.8, 13.7], range: 0–35) were found per child over the entire follow-up period. When both markers were combined, an average of 14.0 *P. vivax* clones were observed per child (CI_95_ [13.0, 15.0], range 0–38).

The average _mol_FOB was 9.4 new *P. vivax* infections per child per year-at-risk by *msp1*F3 (CI_95_ [8.7, 10.0]), 13.8 by MS16 (CI_95_ [12.8, 14.8]) and 15.1 by both markers combined (CI_95_ [14.1, 16.2]). All further analyses were done for both markers combined. In addition, children acquired an average of 5.1 different *P. falciparum msp2* clones during the cohort (CI_95_ [4.6, 5.6], range: 0–19), resulting in a corresponding _mol_FOB of 5.5 new *P. falciparum* infections per child per year-at-risk (CI_95_ [5.0, 6.1]).

**Figure 2:**
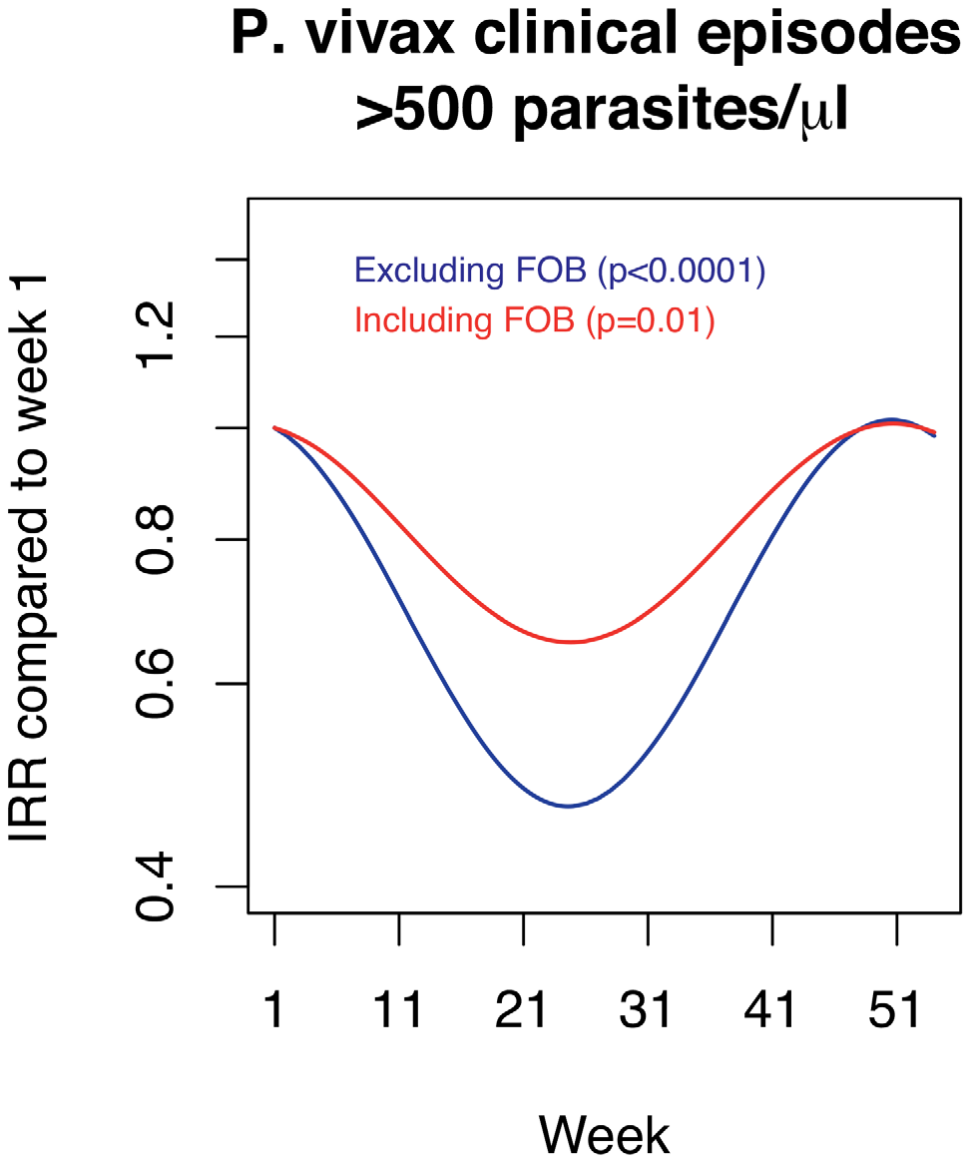
Seasonality of *P.* *vivax* clinical episodes (as compared to the first week of January, where incidence peaked) excluding _mol_FOB (blue) and adjusted for _mol_FOB (red). _mol_FOB accounts for approximately 50% of the seasonal variation in incidence. IRR = incidence rate ratio.

*P. vivax*_mol_FOB showed a very pronounced seasonality (Figure 1, Table 1, *P*<0.0001), peaking in early January (week 1, 17.1 clones/year-at-risk) and was lowest in early July (week 27, 11.3 clones/year-at-risk, Figure 2). _mol_FOB was also significantly lower in 2007 compared to 2006 (incidence rate ratio (IRR) 0.84, CI_95_ [0.77, 0.92], *P* = 0.0002, Table 1, Figure 1). Regular ITN use was associated with a significant reduction in acquisition of new clones (IRR 0.66, CI_95_ [0.56, 0.77], *P*<0.0001, Table 1). Children with antimalarial treatment in the preceding four weeks had a higher _mol_FOB than those that were not treated (IRR 1.24, CI_95_ [1.15, 1.33], *P*<0.0001, Table 1). _mol_FOB did not vary significantly with age (Figure 1, *P* (GLMM) = 0.6). There was significant variation in _mol_FOB between children in a village (*P*<0.001) but not between villages (*P* = 0.3, Table 1).

**Table 1:** Parameter estimates from GLMMs predicting the molecular force of *P. vivax* blood-stage infections (_mol_FOB) and the number of incident clinical episodes of *P. vivax* malaria with density >500 parasites/µl with and without adjustment for _mol_FOB.

	FOB		*P. vivax* >500 episodes		*P. vivax* >500 episodes adjusted for FOB	
	IRR^a^ [CI_95_]	p-value	IRR^a^ [CI_95_]	p-value	IRR^a^ [CI_95_]	p-value
**Fixed effects**						
Age			0.55 [0.46–0.67]	<0.0001	0.52 [0.44–0.62]	<.0001
Sin(week)	0.99 [0.93–1.05]	<0.0001^e^	0.89 [0.77–1.03]	<0.0001^e^	0.94 [0.81–1.09]	<0.01^e^
Cos(week)	1.23 [1.18–1.29]	<0.0001^e^	1.45 [1.25–1.67]	<0.0001^e^	1.24 [1.07–1.44]	<0.01^e^
Average ITN use^b^	0.66 [0.56–0.77]	<0.0001				
Treated^c^	1.24 [1.16–1.33]	<0.0001				
Year 2007	0.84 [0.77–0.92]	0.0002				
FOB∧1/3					1.99 [1.8–2.19]	<0.0001
**Random effects**						
Village	0.007	0.3	0.2	0.0001	0.35	<0.0001
Child	0.2	<0.0001	0.56	<0.0001	0.28	<0.0001
Log likelihood			−669		−551	
AIC^d^			1349		1115	
**Seasonal stats**						
Amplitude	0.21		0.39		0.22	
Month of Peak	early January		early December		early December	
Month of Trough	early July		early June		early June	

a IRR: incidence rate ratio, CI_95_: 95% confidence interval.

b insecticide treated net use: 0% vs 100% use.

c Treated with antimalarials within 28days prior to start of interval.

d Akaike Information Criterium.

e joint p-value for sine and cosine.

### Predictors of clinical *P. vivax* illness

Over the 69 weeks of follow-up, a total of 1134 febrile episodes with parasitemia of any parasite species and any parasite density by light microscopy were observed, resulting in an incidence rate (IR) of 4.60 episodes/year-at-risk [27]. *P. vivax* was the second most common cause of malarial illness, causing 605 episodes (IR = 2.46) with any parasite density (supplementary Table 1). Of these, 391 episodes (IR = 1.59) fulfilled the more specific definition of *P. vivax* malaria (i.e. febrile illness plus *P. vivax* parasitemia >500 parasites/µl) [37]. *P. falciparum* caused slightly more clinical episodes (any density: 630 episodes (IR = 2.56); >2,500 parasites/µl: 472 episodes (IR = 1.92)).

As in earlier analyses [27], age and season were significant predictors of clinical episodes of *P. vivax* malaria (Table 1). The incidence of *P. vivax* malaria decreased log-linearly with age (Figure 1, Table 1, *P*<0.0001) from 2.9 episodes/year-at-risk at 1 year of age to a minimum of 0.6 episodes at 3.5 years of age. It peaked at the beginning of the rainy season (early December, week 49, 1.7 clinical episodes, Figure 1) and was lowest in the early dry season (early June, week 23, 0.8 clinical episodes, Figure 1). Insecticide treated net (ITN) use was not significantly associated with incidence of *P. vivax* malaria. The incidence of *P. vivax* malaria varied significantly between villages (*P*<0.0001) and between children living in the same village (*P*<0.0001).

When _mol_FOB was added to the model (defined as the rate of new clones acquired per year-at-risk), it was highly significantly associated with the incidence of *P. vivax* malaria (Table 1, *P*<0.0001). Adjusting for _mol_FOB resulted in a 45% decrease in seasonal differences in *P. vivax* incidence (Table 1, Figure 2). Contrary to what was observed for *P. falciparum* [23], adding *P. vivax*_mol_FOB did not significantly alter the association of age or ITN use with incidence of clinical *P. vivax* episodes. Comparable results were seen when *P. vivax* episodes with any parasite density were considered (Table S1).

**Figure 3:**
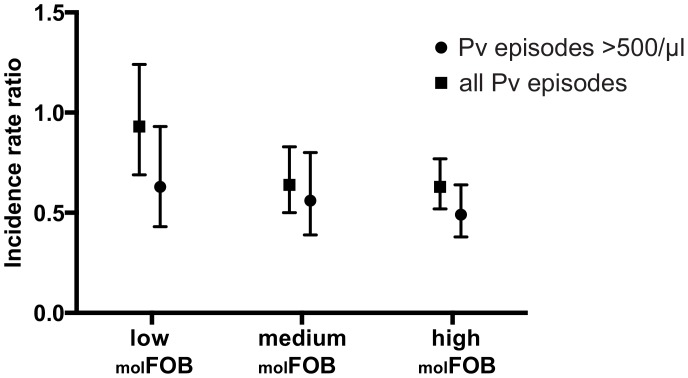
Association of incidence of *P.* *vivax* episodes with age in children with low (_mol_FOB: 0–10.7), medium (_mol_FOB: 10.7–18.0) and high average exposure (_mol_FOB: 18.1–39.0) to *P. vivax* infections. Incidence rate ratio and 95% confidence intervals for changes associated with 1 year increase in age.

In order to determine the association of exposure with the rate of immune acquisition we stratified the cohort in children with high (_mol_FOB: 18.1–39.0/year-at-risk), medium (_mol_FOB: 10.7–18.0/year-at-risk) and low exposure (_mol_FOB: 0–10.7/year-at-risk) based on their average *P. vivax*_mol_FOB. The reduction of incidence of *P. vivax* episodes >500/ul with age was less pronounced in the one third of children with the lowest average _mol_FOB (IRR = 0.63/year increase in age, p = 0.02) than those with the highest exposure (IRR = 0.49/year increase in age, p<0.001) (Figure 3). Even stronger differences were observed in associations of age with all *P. vivax* episodes, where no significant reduction in incidence with increasing age was found for children with low exposure (IRR = 0.93, p = 0.6), but strong reductions in children with medium and high exposure (IRR = 0.50–0.52, p<0.001, Figure 3). These differences in IRR for *P. vivax* episodes of all densities with age were statistically significant for children with low versus medium (p = 0.03) and high exposure (p = 0.02), respectively. Due to overall lower number of episodes with a parasitemia >500 parasites/ul (and thus reduced power), the differences between children with low and medium exposure (p = 0.40) and low and high exposure (p = 0.15) did not reach statistical significance.

## Discussion

By genotyping all blood-stage parasites detected over 16 months of follow-up, this study provides the first direct estimate of the molecular force of *P. vivax* blood-stage infections. Children aged 0.9 to 4.5 years acquired 14.0 *P. vivax* clones per year-at-risk, thus approximately twice as many as *P. falciparum* blood-stage clones [23]. Differences between *P. vivax* and *P. falciparum* were not only evident in the absolute number of infections, but also in the associations between _mol_FOB, incidence of clinical episodes and age. While *P. falciparum*_mol_FOB increased with age and thus paralleled the trend in incidence [23], *P. vivax*_mol_FOB did not change with age, but incidence of disease decreased dramatically over the age range of the cohort.

Minor differences in the typing techniques applied for these two *Plasmodium* species could account for some differences in the estimates. Typing was based on length polymorphic marker genes. Their diversity was high, yet slightly differed: *P. falciparum* was typed using *msp2* (expected heterozygosity *H*_E_ = 0.933) [38], while *msp1*F3 (*H*_E_ = 0.881) and MS16 (*H*_E_ = 0.978) were used for *P. vivax* typing [28]. For all three markers, we previously determined the clone detectability, i.e. the proportion of clones detected in both of two bleeds collected 24 hours apart. Detectability differed between markers: 79% for *Pfmsp2*, 61% for *Pv*MS16 and 73% for *Pvmsp1*F3 [30]. The overall diversity of the *Pfmsp2* marker was therefore intermediate to that of the two *P. vivax* markers, while *Pfmsp2* detectability was highest.

The most obvious difference in typing strategies was that our analysis of *P. vivax* was based on two loci with the combined _mol_FOB determined from maximal number of alleles per sample observed by any marker. Thus, the ability to detect clones of both *P. vivax* markers combined was higher than by the single *P. falciparum* marker.

To assess the effect of the use of 2 markers, *P. vivax*_mol_FOB was also calculated using a single marker only. The average _mol_FOB was 8.7 and 12.8 clones/year-at-risk for *msp1*F3 and MS16, respectively. Both of these values are substantially higher than the 5.5 clones/year-at-risk detected by *Pfmsp2* genotyping. It is worth noting that a 1.6 times higher _mol_FOB was obtained with *P. vivax* marker *msp1*F3 compared to *P. falciparum* despite its lower diversity and detectability. Therefore, the differences in allelic diversity and detectability of genotyping markers can not account for the large differences between *P. vivax* and *P. falciparum*_mol_FOB.

Given imperfect detectability and resolution of markers plus infrequent sampling, our estimates of _mol_FOB of both species were likely underestimates of the true burden and complexity of *Plasmodium* spp. infection. However, for 97.5% (1638/1680) of all regular follow-up bleeds (except baseline and the final round), two paired samples taken 24 hours apart were available, thus the probability of missing a clone in both of two paired samples dropped to 0.07 for *P. vivax msp1*F3, 0.15 for *P. vivax* MS16 and 0.044 for *P. falciparum msp2* [30] (assuming independence in the chance to detect a clone from any one sample). In addition, clones detected during passive case detection between regular visits were also included, further reducing the chance of missing a clone during an 8-week interval. This suggests that the estimates of _mol_FOB were biased downwards as a result of imperfect detectability only by between 6 and 13% for *P. vivax* and 4% for *P. falciparum*.

In PNG, *P. vivax* and *P. falciparum* are transmitted by the same mosquito vectors. While no entomological studies were conducted in parallel with this cohort, earlier studies in different PNG lowland populations reported comparable sporozoite rates for *P. falciparum* and *P. vivax* [6], [39], [40]. However, the likelihood that individuals become infected with multiple *P. vivax* clones by a single mosquito bite is high, because 75% of all *P. vivax* positive individuals from our cohort carried multiple clone infections [28]. It is therefore likely that a mosquito takes up different *P. vivax* gametocyte clones during a blood-meal, resulting in sexual recombination and the transmission of a genetically diverse inoculum to a new host. This contrasts to only 33% of multi-clone infections in the *P. falciparum* positive children (Schoepflin, Mueller & Felger unpublished results).

*P. vivax* parasites detected in the blood stream do not always derive from a recent mosquito bite; they can also result from relapsing hypnozoites. New Guinean *P. vivax* strains were reported to relapse rapidly, and 63% of 1 to 5 year old children had a recurrent parasitemia within six weeks after treatment of blood-stage parasites [41]. Two thirds of those recurrent infections (all post day 28) were of a different genotype [42]. Although genotyping cannot differentiate between true new infections and relapses of an unrelated genotype, *P. vivax* relapses likely contribute significantly to the higher _mol_FOB. Evidence for this comes from a recent cohort of Papua New Guinean children aged 1 to 5 years, in which the contribution of relapses to the burden of infection was assessed directly by randomising one third of children to receive anti-hypnozoite drug therapy with a 14 day course of high-dose primaquine. While the primaquine treatment was not 100% efficacious, it did nevertheless result in a 34–57% reduction in the incidence of new *P. vivax* infections [43].

As a consequence of relapses, _mol_FOB measures different processes in *P. vivax* and *P. falciparum*. *P. falciparum*_mol_FOB is directly linked to transmission intensity in a given interval, whereas _mol_FOB of *P. vivax* represents a composite measure of both transmission intensity and frequency and genetic complexity of relapsing parasites (some of which may have been acquired months or years earlier). *P. vivax*_mol_FOB is therefore not a direct measure of _mol_FOI (i.e. the number of new parasites acquired by the human host), but the difference between the two measures is small if the total follow-up period is substantially longer than the average relapse frequency. In the Southwest Pacific, *P. vivax* infections are thought to relapse very rapidly (i.e. within a few weeks [31]), thus *P. vivax*_mol_FOB is a good surrogate marker for _mol_FOI if calculated over the entire 16 months of follow-up.

Interestingly, *P. vivax*_mol_FOB did not change with age, while *P. falciparum*_mol_FOI was strongly age dependent and increased from 3 to 8 clones per year-at-risk over the age range of 1–4.5 years of this cohort [28]. As a consequence, _mol_FOI largely explained the age trend in *P. falciparum* incidence (increasing from 1 episode per year in children one year of age to 2.5 in children three years of age) [23]. In contrast, *P. vivax*_mol_FOB did not explain the decrease in *P. vivax* incidence from over 3 episodes per year in children one year of age to less than 1 episode in children older than three years.

The age shift in incidence in clinical disease caused by *P. vivax* and *P. falciparum* observed in this study – i.e. *P. vivax* incidence peaking in younger children - parallels earlier findings in Papua New Guinea [6], [10], [29] and other regions where both species are co-endemic [7]–[9]. This indicates a rapid acquisition of immunity to *P. vivax* in individuals with life-long exposure to both *P. falciparum* and *P. vivax*. As immunity to malaria builds up gradually and is thought to be strain-specific [4], [5], it is likely that the number of distinct infections acquired over an individual's lifetime is a major driving force for acquisition of immunity [20].

In their first years of life, the children in our cohort were estimated to have acquired three times more genetically distinct *P. vivax* than *P. falciparum* infections. In both species a higher _mol_FOB was itself associated with a significant increase in incidence of clinical disease. However, a more rapid decrease in incidence of *P. vivax* malaria was observed in children exposed to high compared to low levels of *P. vivax* infections (Figure 3). Although notable against *P. vivax* episodes with >500 parasites/µl, this effect of exposure on age-specific risk of *P. vivax* malaria was more pronounced when all *P. vivax* episodes (irrespective of parasitaemia) were considered. While the incidence of *P. vivax* malaria >500 parasites/µl decreased, even in children with low exposure, the incidence of *P. vivax* of any density did not change significantly with age in the children with the low exposure. This indicates that, in children with low exposure, those >3 years acquired some immunity against high-density clinical *P. vivax* episodes, but not against those associated with low levels of parasitaemia. Together these observations suggest that high _mol_FOB, and consequently the overall genetic diversity to which children were exposed in early childhood, contribute substantially to the rapid acquisition of clinical immunity to *P. vivax* across the entire age range [27].

The high exposure to *P. vivax* may even be sufficient for children to acquire a certain degree of immunity against *P. vivax* infection. With at least some malaria vectors, biting rates increase with host body size [44]. Therefore, as children grow older, their exposure to malarial infection also increases, as supported by the strong age-dependence of *P. falciparum*_mol_FOI [28]. The lack of an association of *P. vivax*_mol_FOB with age suggests that, in older children, some of the *P. vivax* sporozoites transmitted by mosquitoes do not succeed in establishing detectable blood stage infections.

### Conclusions

We propose that the high number of genetically distinct *P. vivax* blood stages infections acquired in the first 4 years of life (as measured by _mol_FOB) is a significant contributor to the rapid acquisition of immunity against clinical *P. vivax* malaria. Albeit less closely linked to transmission (i.e. force of infection (FOI)) than in *P. falciparum* [23], _mol_FOB is nevertheless a measure of individual exposure to *P. vivax* blood-stage infection and is significantly linked to the observed burden of *P. vivax* malaria. As such, it could be used as both a surrogate maker for exposure and as a parameter for monitoring the impact of antimalarial interventions.

## Supporting Information

Checklist S1 STROBE checklist.
(DOC)

Table S1 Parameter estimates from GLMMs predicting the number of incident clinical episodes of *P. vivax* malaria with any parasites density with and without adjustment for _mol_FOB.
(DOCX)
