# Supplementary material for: A High Force of Plasmodium vivax Blood-Stage Infection Drives the Rapid Acquisition of Immunity in Papua New Guinean Children
Source: PLoS Negl Trop Dis. 2013 Sep 5;7(9):e2403. doi: 10.1371/journal.pntd.0002403 (PMC3764149; doi:10.1371/journal.pntd.0002403)
Supplement: Checklist S1 — STROBE checklist. (DOC) [file pntd.0002403.s001.doc]

**Checklist S1: STROBE Checklist**

STROBE Statement—Checklist of items that should be included in reports of ***cohort studies***

|  | Item No | Recommendation |
| --- | --- | --- |
| **Title and abstract** | 1 | (*a*) Done |
|  |  | (*b*) Done |
| Introduction | | |
| Background/rationale | 2 | Introduction, Paragraphs 2-6 |
| Objectives | 3 | Introduction, Paragraph 7 |
| Methods | | |
| Study design | 4 | Methods, Paragraph 2 |
| Setting | 5 | Methods, Paragraph 2, reference 27 |
| Participants | 6 | (*a*) Methods, Paragraph 2, reference 27 |
|  |  | (*b*) |
| Variables | 7 | Methods, Paragraph 2 and section “Data analysis”, reference 27 |
| Data sources/ measurement | 8* | Methods, Section “Data Analysis” |
| Bias | 9 | Reference 27 |
| Study size | 10 | Reference 27 |
| Quantitative variables | 11 | Methods, Section “Data Analysis” |
| Statistical methods | 12 | (*a*) Methods, Section “Data Analysis” |
|  |  | (*b*) Methods, Section “Data Analysis”, paragraph 3 |
|  |  | (c) Methods, paragraph 6, references 27 & 30 |
|  |  | (*d*) Reference 27 |
|  |  | (*e*) Not applicable |
| Results | | |
| Participants | 13* | (a) Results, paragraphs 1 |
|  |  | (b) Reference 27 |
|  |  | (c) Not necessary |
| Descriptive data | 14* | (a) Methods, Paragraph 2, reference 27 |
|  |  | (b) Reference 27 |
|  |  | (c) Reference 27 |
| Outcome data | 15* | Results, paragraphs 1-3 |
| Main results | 16 | (*a*) Done in results section |
|  |  | (*b*) Done in results section (high/medium/low exposure) |
|  |  | (*c*) Not necessary |
| Other analyses | 17 | Results, last paragraph (high/medium/low exposure) |
| Discussion | | |
| Key results | 18 | Discussion, paragraph 1 |
| Limitations | 19 | Discussion, paragraph 2-5 |
| Interpretation | 20 | Discussion, paragrpahs 10-12 and Conclusions |
| Generalisability | 21 | All Discussion |
| Other information | | |
| Funding | 22 | Submitted separate to manuscript on PLoS NTDs submission page |

*Give information separately for exposed and unexposed groups.
