# Supplementary material for: A High Force of Plasmodium vivax Blood-Stage Infection Drives the Rapid Acquisition of Immunity in Papua New Guinean Children
Source: PLoS Negl Trop Dis. 2013 Sep 5;7(9):e2403. doi: 10.1371/journal.pntd.0002403 (PMC3764149; doi:10.1371/journal.pntd.0002403)
Supplement: Table S1 — Parameter estimates from GLMMs predicting the number of incident clinical episodes of P. vivax malaria with any parasites density with and without adjustment for molFOB. (DOCX) [file pntd.0002403.s002.docx]

**Supplementary Table S1**: Parameter estimates from GLMMs predicting the number of incident clinical episodes of *P. vivax* malaria with any parasites density with and without adjustment for _mol_FOB.

|  |  |  | |  |  |  | |  |
| --- | --- | --- | --- | --- | --- | --- | --- | --- |
|  |  | ***P. vivax* any density** | | |  | ***P. vivax* any density**  **adjusted for FOB^b^** | | |
|  |  | **IRR^a^** | | **p - value** |  | **IRR^a^** | | **p - value** |
|  |  |  |  |  |  |  |  |  |
|  |  |  | |  |  |  | |  |
| Age (at interval) |  | 0.72 [0.62, 0.83] | | <0.0001 |  | 0.69 [0.60, 0.78] | | 0.3 |
|  |  |  | |  |  |  | |  |
| Sin (week) |  | 0.88 [0.78, 0.99] | | <0.0001^c^ |  | 0.92 [0.82, 1.03] | | <0.0001^c^ |
| Cos (week) |  | 1.48 [1.31, 1.66] | |  |  | 1.28 [1.14, 1.45] | |  |
|  |  |  | |  |  |  |  |  |
| FOB^1/3 |  | NA | | NA |  | 1.89 [1.74, 2.04] | | < 0.0001 |
|  |  |  | |  |  |  | |  |
| Random effects: |  |  | |  |  |  | |  |
| *Village* |  | 0.08 | | < 0.01 |  | 0.14 | | <0.001 |
| *Child within village* |  | 0.33 | | < 0.001 |  | 0.11 | | <0.01 |
|  |  |  | |  |  |  | |  |
| Log likelihood |  | -843 | |  |  | -683 | |  |
| AIC^d^ |  | 1701 | |  |  | 1382 | |  |
|  |  |  | |  |  |  | |  |
| Seasonal effects |  |  | |  |  |  | |  |
| Amplitude |  | 0.41 | |  |  | 0.26 | |  |
| Peak (week) |  | early December | |  |  | early December | |  |
| Trough (week) |  | early June | |  |  | early June | |  |

^a^ IRR: incidence rate ratio, CI_95_**:** 95% confidence interval

^b^ Force of infection (# new clones per year-at-risk), cube-root transformed.

^c^ combined p-value for sin and cos

^d^ Akaike Information Criterium
